# Mucous Membrane Pemphigoid with Tracheal Involvement

**DOI:** 10.1155/2016/5749784

**Published:** 2016-02-03

**Authors:** Arash Minaie, Salim R. Surani

**Affiliations:** ^1^Bay Area Medical Center, University of North Texas, 7121 S. Padre Island Drive, Corpus Christi, TX 78412, USA; ^2^University of North Texas, 7121 S. Padre Island Drive, Corpus Christi, TX 78412, USA

## Abstract

34-year-old African American female with history of pemphigoid presented with hemoptysis. Patient was found to have mucous membrane pemphigoid involving the oropharynx and extending to trachea, till just above main stem carina. Four other cases described mucosal pemphigoid involving the trachea. We hereby present a brief review of current consensus on management of mucous membrane pemphigoid with airway involvement.

## 1. Introduction

Mucous membrane pemphigoid is a chronic inflammatory autoimmune disease process that is characterized by subepithelial blistering, primarily of the mucosa, with linear deposition of Immunoglobulin G (IgG), Immunoglobulin A (IgA), and Complement 3 (C3) at the basement membrane [1]. Unfortunately, scarring and loss of function can be major sequelae. Affected areas, in order of frequency, include oral, ocular, nasal, nasopharyngeal, anogenital, skin, laryngeal, and esophageal involvement [1–3].

While there have been a number of case reports of mucous membrane pemphigoid, less than a handful of the cases involve the trachea [4–7]. This paper aims to increase the awareness of tracheal involvement due to mucous membrane pemphigoid and its management.

## 2. Case Report

**Figure 1:**
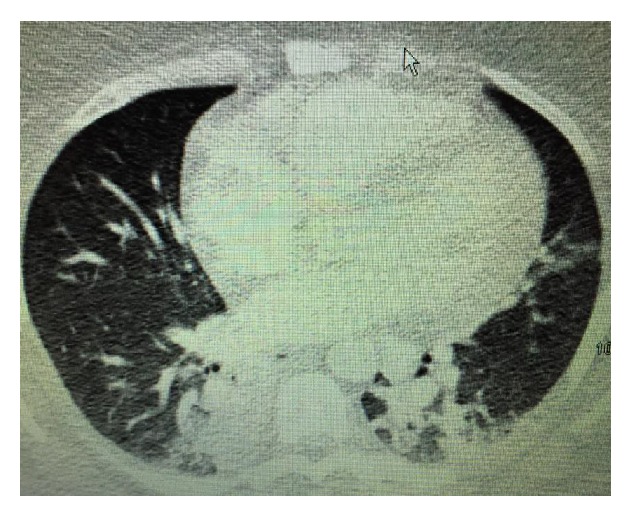
CT of chest without contrast displaying bilateral lower lobes, right greater than left.

**Figure 2:**
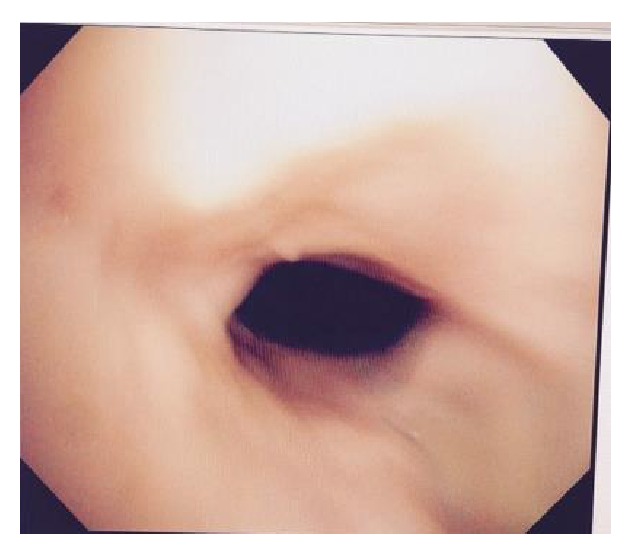
Trachea directly below vocal cords prior to jet of saline showing large amount of abnormal edematous greyish white mucosa.

**Figure 3:**
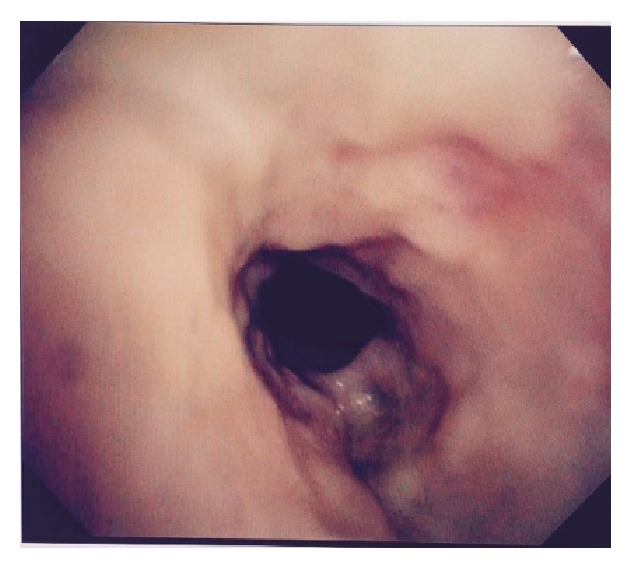
Area below vocal cord following administration of saline jet with sloughing of tracheal mucosa.

**Figure 4:**
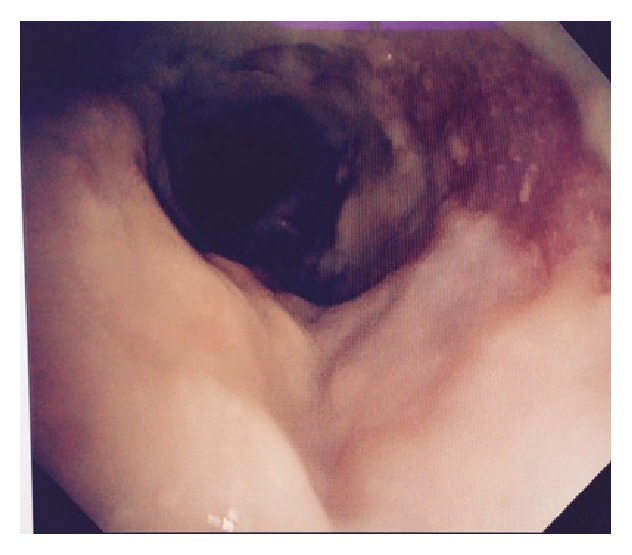
Closer look at area of ulceration following administration of saline.

**Figure 5:**
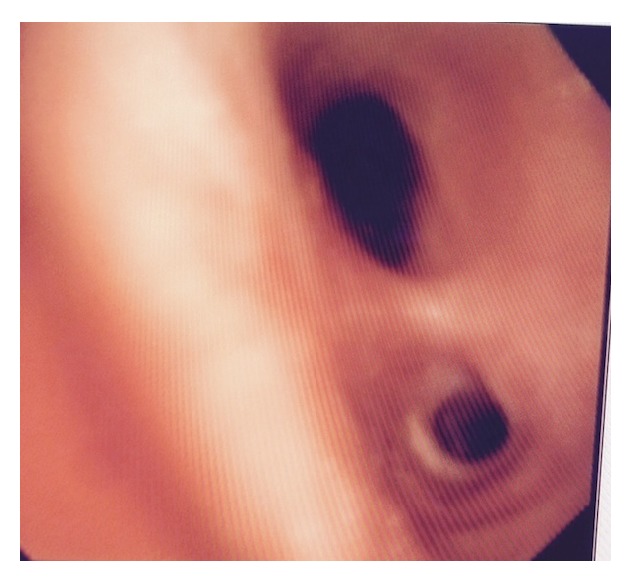
Abrupt end to whitish grey mucosa directly following carina.

A 34-year-old African American female patient with a history of obstructive sleep apnea, Graves disease, and mucocutaneous pemphigoid with corneal and oropharynx involvement presented to our emergency department (ED). She was diagnosed by the biopsy of her corneal lesion and immunofluorescence studies which was consistent with the diagnosis of mucous membrane pemphigoid. In past she was treated with methotrexate and prednisone. She developed pneumonia while being on methotrexate, which was then stopped. Later she was given rituximab due to her progression of disease for 3 months. She presented to our emergency department (ED) with a 1-week history of malaise, lethargy, nausea, and productive cough with thick yellow sputum and approximately 25 mL of hemoptysis daily. In the emergency ED, the patient was found to have conversational dyspnea with severe wheezing. She was placed on oxygen via nasal cannula at 5 L/min and anticholinergic and antimuscarinic medication via nebulizer therapy for bronchodilatation, followed by intravenous methylprednisolone, vancomycin, and piperacillin/tazobactam. Arterial blood gas showed a pH of 7.45, partial pressure of carbon dioxide (CO_2_) of 44.3 mm Hg, and partial pressure of oxygen (PaO_2_) of 57.8 mmHg. She was also found to have a white blood cell count of 28.8·10^9^/liter and a hemoglobin level of 12.2 g/dL. Chest X-ray demonstrated cardiomegaly with prominence of pulmonary vessels. Computed tomography of the chest demonstrated infiltrates in bilateral lower lobes, right greater than left, consistent with pneumonia (Figure 1). She was subsequently admitted to the hospital. Patient underwent bronchoscopy for persistent hemoptysis, which revealed mucosal ulceration in the oral cavity consistent with mucous membrane pemphigoid. The entire trachea below the level of the vocal cord showed mucosa to be very abnormal, and what would normally be pink tracheal mucosa was replaced with thick whitish gray mucosa (Figure 2), which peeled and sloughed off easily with a gentle saline jet (Figures 3 and 4). The whitish gray mucosa abruptly stopped at the carina as demonstrated in Figure 5. The patient sputum culture grew methicillin resistant* Staphylococcus aureus* (MRSA), which responded to the treatment with vancomycin.

## 3. Discussion

Mucous membrane pemphigoid is a spectrum of bullae forming diseases that is characterized by deposition of subepithelial immunoglobulin with involvement of oral, ocular, nasal, nasopharyngeal, anogenital, skin, laryngeal, and esophageal basement membranes [1–3, 8]. This spectrum has been labeled with different names as cicatricial pemphigoid, benign mucous membrane pemphigoid, oral pemphigoid, desquamative gingivitis, and ocular cicatricial pemphigoid [8].

Our review of the literature revealed only four similar cases with tracheal involvement of bullae. In the study by Kato et al., the patient had skin, eye, oropharyngeal, and tracheal involvement to the initial part of the main stem bronchi [4]. What was described was very similar to our observation in the abrupt ending of lesions close to the carina; however, the lesions do extend to the beginning of the main stem bronchi in their description. de Carvalho et al. described the case of a 20-year-old woman with tracheal involvement [5]. Yasuda et al. described a case of a 49-year-old female who developed skin manifestations and a few bullae next to the endotracheal tube itself [6]. Much like our case, all three of these cases described involvement primarily restricted to the large airways that did not go past the beginning of the main stem bronchi. Furthermore, all cases reported with involvement of the trachea seem to be in females, but with no correlation to race [4–6].

Mucous membrane pemphigoid is generally diagnosed with a combination of clinical and immunopathological findings. The biopsy is generally made at the edge of the lesion, and direct immunofluorescence staining shows a linear deposition of IgG, IgA, or C3 along the basement membrane [8]. Treatment is generally divided into two categories of high-risk patients and low-risk patients based on site, severity, and rapidity of progression. Two out of the four cases reported have resulted in fatalities [4–7]. In the event of tracheal involvement, the likelihood of compromise of the entire airway makes these cases particularly high risk.

The treatment regimen of choice may include one of the following: prednisone 1–1.5 mg/kg per day, cyclophosphamide 1-2 mg/kg per day, or azathioprine 1-2 mg/kg per day (patient may need bridging due to a 4–8-week lag time for full effect) [8]. Due to the deposition of IgG and IgA along the basement membrane, rituximab has been recently shown to have some promise. A small number of patients had successful remission on treatment with rituximab; however, all of those patients had relapses. Further studies need to be conducted to compare rituximab to other therapies and define the optimal protocol to be used for treatment of mucous membrane pemphigoid [9, 10]. Additionally, patients with ophthalmic involvement exhibited a reduction in IgM following administration of rituximab but still had high levels of IgA, which can be a concern in IgA-predominant variants [11, 12].

## 4. Conclusions

There are few cases of tracheal involvement in patients with pemphigoid. Hemoptysis or respiratory compromise in a patient with mucous membrane pemphigoid should prompt the clinician to investigate whether the patient has tracheal involvement and consider intensification of therapy. More research is needed to compare targeted humoral immunosuppressive therapy with corticosteroids or disease modifying antirheumatic drugs (DMARDs) such as cyclophosphamide or azathioprine for both induction and maintenance of remission.
